# Syringaresinol attenuates Tau phosphorylation and ameliorates cognitive dysfunction induced by sevoflurane in aged rats

**DOI:** 10.1093/jnen/nlae026

**Published:** 2024-04-15

**Authors:** Simin Zheng, Yunpeng Teng, Hongtao Liu, Jiaxuan He, Shaobo Zhang, Hongfei Xiong

**Affiliations:** Department of Anesthesiology, The Second Affiliated Hospital of Xi’an Jiaotong University, Xi’an, Shannxi, China; Department of Anesthesia and Comfort Health Center, Xi’an International Medical Center Hospital, Xi’an, Shannxi, China; Department of Anesthesiology, The Second Affiliated Hospital of Xi’an Jiaotong University, Xi’an, Shannxi, China; Department of Anesthesia and Comfort Health Center, Xi’an International Medical Center Hospital, Xi’an, Shannxi, China; Department of Anesthesiology, The Second Affiliated Hospital of Xi’an Jiaotong University, Xi’an, Shannxi, China; Department of Anesthesia and Comfort Health Center, Xi’an International Medical Center Hospital, Xi’an, Shannxi, China

**Keywords:** Cognitive dysfunction, Neuroprotection, Sevoflurane, SIRT1 pathway, Syringaresinol

## Abstract

Cognitive dysfunction following anesthesia with agents such as sevoflurane is a significant clinical problem, particularly in elderly patients. This study aimed to explore the protective effects of the phytochemical syringaresinol (SYR) against sevoflurane-induced cognitive deficits in aged Sprague-Dawley rats and to determine the underlying mechanisms involved. We assessed the impact of SYR on sevoflurane-induced cognitive impairment, glial activation, and neuronal apoptosis through behavioral tests (Morris water maze), immunofluorescence, Western blotting for key proteins involved in apoptosis and inflammation, and enzyme-linked immunosorbent assays for interleukin-1β, tumor necrosis factor-α, and interleukin-6. SYR treatment mitigated sevoflurane-induced cognitive decline, reduced microglial and astrocyte activation (decreased Iba-1 and GFAP expression), and countered neuronal apoptosis (reduced Bax, cleaved-caspase3, and cleaved-PARP expression). SYR also enhanced Sirtuin-1 (SIRT1) expression and reduced p-Tau phosphorylation; these effects were reversed by the SIRT1 inhibitor EX527. SYR exerts neuroprotective effects on sevoflurane-induced cognitive dysfunction by modulating glial activity, apoptotic signaling, and Tau phosphorylation through the SIRT1 pathway. These findings could inform clinical strategies to safeguard cognitive function in patients undergoing anesthesia.

## INTRODUCTION

Postoperative cognitive dysfunction (POCD) is a cognitive disorder that occurs after anesthesia and surgery, affecting patients’ attention, concentration, memory, information processing, executive functions, visual-spatial abilities, and psychomotor speed. POCD not only severely impacts the quality of daily life of patients but also extends hospital stays and increases medical costs, imposing significant psychological and financial burdens on patients and their families. Studies indicate that approximately 12% of patients who undergo noncardiac surgery and have previously demonstrated good cognitive abilities exhibit symptoms of POCD postsurgery (1). Sevoflurane, one of the most commonly used volatile anesthetics, known for its rapid induction and excellent controllability, has been shown to potentially cause neuropathological changes and long-term cognitive impairments in humans and animals upon repeated exposure. The neurotoxic effects of sevoflurane may be mediated through mechanisms such as neuroinflammation, neurotransmitter imbalance, and/or reductions in brain-derived neurotrophic factor (BDNF) concentrations (1).

Sirtuin-1 (SIRT1), a member of the Sirtuin family, is a nicotinamide adenine dinucleotide (NAD+)-dependent class III histone deacetylase found in various tissues, including the brain. SIRT1 regulates numerous cellular processes by binding to and deacetylating various targets, such as nuclear factor kappa B (NF-κB). The activity of SIRT1 has been shown to have neuroprotective properties in neurodegenerative and psychiatric disorders, with a noted decrease in its expression levels in POCD. Anesthesia and surgery have been observed to cause morphological changes in microglia, increased synaptic phagocytosis, dendritic spine loss, and cognitive deficits that can be mitigated by the administration of SIRT1 agonists (2). Tau protein, as one of the microtubule-associated proteins, plays a critical role in cognitive dysfunction. Abnormal hyperphosphorylation of Tau is closely associated with the neuropathogenesis of cognitive dysfunction (3). Studies suggest that activation of SIRT1 inhibits acetylation and phosphorylation of Tau in aged rats, thereby reducing neurodegeneration and cognitive impairments induced by sevoflurane (4).

Syringaresinol (SYR), extracted from various parts of plants such as ginseng berries, magnolia, and cinnamon, has been extensively proven to possess anti-inflammatory and antioxidant activities (5). SYR significantly improves cardiac function in cecal ligation and puncture model mice, alleviates myocardial injury, and has been shown to activate SIRT1 (6). Additionally, SYR alleviates neuropathic pain symptoms induced by oxaliplatin by inhibiting the inflammatory response of spinal microglia (7) and suppresses excitatory synaptic transmission in the hippocampus and pentylenetetrazole-induced epileptic activity through presynaptic mechanisms (8). However, the role and mechanism of SYR in sevoflurane-induced cognitive dysfunction remain unclear. The aim of this study was to investigate the modulation of SYR in sevoflurane-induced cognitive dysfunction.

## MATERIALS AND METHODS

### Model induction and group designation

We used 24 aged male Sprague-Dawley rats (24 months old, 240–300 g) from Xi’an International Medical Center Hospital Laboratory Animal Center, with ethical approval from the Xi’an International Medical Center Hospital Ethics Committee. Rats were housed in acrylic cages under controlled conditions (12-hour light/dark cycle, 21 ± 1°C, 45%–65% humidity), with free access to food and water. Behavioral tests were conducted in a temperature- and humidity-controlled lab. The rats were divided into 2 main groups: the sevoflurane group (Sev) and the control group. The Sev group received 2.6% sevoflurane anesthesia for 4 hours, delivered at 2 L/min with 30% oxygen. The control group inhaled carrier gas without sevoflurane for the same duration. Further, all rats were assigned into 4 subgroups (6 rats each): control, 20 mg/kg SYR orally, sevoflurane anesthesia, and a combination of SYR and sevoflurane. Behavioral assessments were conducted postanesthesia. After the assessment, the animals were euthanized for hippocampal tissue extraction for molecular analysis. In the third experiment phase, the anesthetized rats were divided into 4 groups: control, SYR, Sev, and Sev+SYR. Additionally, to explore the mechanisms involved, we used EX527, a SIRT1 inhibitor, to study the pathway regulated by SYR.

### Morris water maze test

The Morris water maze (MWM) test was slightly modified from previous methods to assess the spatial memory and learning abilities of rats. The water temperature was maintained at 22 ± 1°C to prevent floating. A circular escape platform (diameter 22 cm) was placed 0.5–1 cm below the water surface in 1 of the 4 quadrants of the maze. After 5 days of training, the rats underwent reference memory tests at 3 time points, each consisting of 4 trials with a 15-minute interval. At the second and third time points, the platform was removed from the water to measure spatial and working memory. The time each rat took to find the platform was recorded as the escape latency period. After the test, the rats were placed in a heated cage for a short rest before being returned to their original cage. Following the previous description, open field tests were conducted after anesthesia. The central area, accounting for 66% of the field, was defined as the “center.” The time spent in the center was recorded as a parameter to assess antianxiety behavior.

### Enzyme-linked immunosorbent assay

All rats were euthanized after the behavioral tests on day 4. Hippocampal tissue was immediately dissected on ice and homogenized in saline with ultrasonication. This was followed by centrifugation at 10 000*g* for 15 minutes at 4°C. Levels of interleukin-1β (IL-1β), tumor necrosis factor-α (TNF-α), and interleukin-6 (IL-6) were determined using enzyme-linked immunosorbent assay (ELISA) kits (eBioscience, San Diego, CA) as per the manufacturer’s instructions. Concentrations of IL-1β, TNF-α, and IL-6 in the plasma were also measured by ELISA.

### Hematoxylin and eosin staining

Hematoxylin and eosin (H&E) staining was employed to assess hippocampal tissue damage. Stained sections were observed at 400× magnification under an Olympus microscope to observe pathological changes.

### Quantitative PCR

Total RNA from hippocampal tissues was extracted by homogenization using TRIzol reagent (Invitrogen, Waltham, MA). Transcriptional amplification was then performed using the iScript cDNA Synthesis Kit (Bio-Rad, Hercules, CA). qPCR amplification was conducted using iTaq Universal SYBR Green Supermix (Bio-Rad) and gene-specific primers. Relative expression levels of each gene were calculated using the 2^−ΔΔCt^ method and normalized against GAPDH. Primer sequences are listed in the Table 1.

**Table 1.** The primers of qRT-PCR

Gene	Primer sequences
IL-1β	Forward: 5′-GACCTGTTCTTTGAGGCTGACA-3′
	Reverse: 5′-CTCATCTGGACAGCCCAAGTC-3′
IL-6	Forward: 5′-TAGTCCTTCCTACCCCAACTTCC-3′
	Reverse: 5′-TTGGTCCTTAGCCACTCCTTC-3′
TNF-α	Forward: 5′-GACCCTCACACTCAGATCATCTTCT-3′
	Reverse: 5′-TGCTACGACGTGGGCTACG-3′
GAPDH	Forward: 5′-CATGGCCTTCCGTGTTCCTA-3′
	Reverse: 5′-GCGGCACGTCAGATCCA-3′

### Immunofluorescence

Immunofluorescence staining was performed on hippocampal tissue sections to observe and quantify the expression of specific proteins. Initially, brain sections were rinsed with 0.01 M phosphate-buffered saline (PBS) and then incubated with a 5% bovine serum albumin blocking buffer to prevent nonspecific binding. Subsequently, the sections were incubated overnight at 4°C with primary antibodies. After primary antibody incubation, the sections were washed 3 times with PBS and then incubated with biotinylated secondary antibodies conjugated with Alexa Fluor 488 or 594 for 2 hours at room temperature. The primary antibodies used were: Iba-1 (1:1500) (Cat no. 17198; Cell Signaling Technology, Danvers, MA), and GFAP (1:1500) (Cat no. 12389; Cell Signaling Technology). During immunofluorescence staining, the slides were washed 3 times and subsequently covered with mounting medium containing 4,6-diamidino-2-phenylindole (DAPI) (Sigma-Aldrich, F6057, St Louis, MO). Immunofluorescence microscopy images were acquired using a confocal microscope (model FluoView FV 1000, manufacturer Olympus, Tokyo, Japan). To ensure the reliability of the results, at least 5 images were taken of brain sections from each animal and repeated 6 times for all measurements.

### TdT-mediated dUTP nick end labeling staining

The TdT-mediated dUTP nick end labeling (TUNEL) staining method was utilized to identify cellular damage in hippocampal tissue, a widely used technique for detecting DNA fragmentation and apoptosis. Staining was carried out according to the standard operating procedure provided by Roche, effectively labeling damaged cells. Stained tissue sections were observed and photographed under a fluorescence microscope (U-HGLGPS, Olympus) at a 40× magnification. Subsequently, the number of TUNEL-positive cells was counted using ImageJ software (Version 1.8.0). For quantitative analysis, the data were expressed as the number of TUNEL-positive cells per square millimeter.

### Western blotting

Hippocampal tissues were homogenized in RIPA lysis buffer. Proteins were separated using SDS-PAGE gel and then transferred to PVDF membranes (Millipore, Burlington, MA). The membranes were blocked with 5% milk and then incubated with specific primary antibodies, including anti-Iba-1 (1:1500) (Cat no. 17198; Cell Signaling Technology), anti-GFAP (1:1500) (Cat no. 12389; Cell Signaling Technology), anti-Bax (Cat no. 2772; Cell Signaling Technology), anti-Bcl-2 (Cat no. 15071; Cell Signaling Technology), anticleaved-caspase3 (Cat no. 9661; Cell Signaling Technology), anticleaved-PARP (Cat no. 9541; Cell Signaling Technology), anti-SIRT1 (Cat no. 2028; Cell Signaling Technology), anti-p-Tau (Cat no. 12885; Cell Signaling Technology), and anti-GAPDH (Cat no. 2118; Cell Signaling Technology). The membranes were washed with TBST, followed by incubation with HRP-conjugated secondary antibodies (Cat no. 58802, 7074; Cell Signaling Technology). Protein bands were visualized using the ECL system as per the manufacturer’s instructions. Relative protein expression levels were quantitatively analyzed using ImageJ software.

### Statistical analysis

Data analysis was conducted using GraphPad Prism 6.0 software. Behavioral study data at different time points postsevoflurane anesthesia were analyzed using repeated-measures 2-way analysis of variance (ANOVA). Other data were subjected to 1-way ANOVA prior to Bonferroni correction. All experiments were replicated 3 times, and the results are presented as mean ± SD of the mean.

## RESULTS

### SYR attenuates sevoflurane-induced cognitive dysfunction in rats

**Figure 1.**
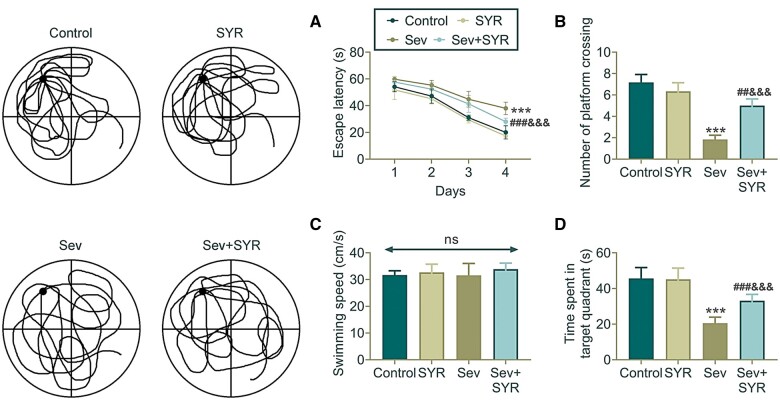
Comparative analysis of spatial learning in rodents subjected to different treatments using an MWM. **(A)** Escape latency trends over a period of 4 days are plotted, showing a decrease in time for the Sev+SYR group. Representative tracings are shown on the left. **(B)** Frequency of platform crossings, which serves as a measure of memory, is displayed with the Sev+SYR group exhibiting fewer crossings. **(C)** No significant difference in swimming speed among groups suggests equal locomotive function. **(D)** Time spent in the target quadrant is graphed, with the Sev+SYR group spending more time, indicating better memory retention; n = 6, ***p < .001 compared to the control group, ^###&&&^p < .001 compared to the SYR and Sev group.

Potential therapeutic effects of SYR on cognitive impairment induced by Sev in rats were evaluated using the MWM (Fig. 1). Compared to the control group, the Sev group had a significantly increased escape latency while the Sev + SYR group had a significantly reduced escape latency, approaching the level of the control group. This indicates that SYR can effectively alleviate cognitive impairment caused by Sev. The number of platform crossings further supported the protective effect of SYR, with the Sev+SYR group crossing significantly more than the Sev group. The time spent in the target quadrant was significantly lower in the Sev group compared to the control group, but was significantly higher in the Sev + SYR group, further demonstrating the effectiveness of SYR in improving memory impairment induced by Sev. Moreover, the measurement of swimming speed showed no significant difference between all groups, indicating that the improvement in cognitive function was not due to changes in motor ability. These results suggest that SYR can restore memory capabilities by alleviating cognitive dysfunction induced by Sevoflurane, and that the mechanisms of action warrant further investigation.

### SYR alleviates hippocampal tissue damage

**Figure 2.**
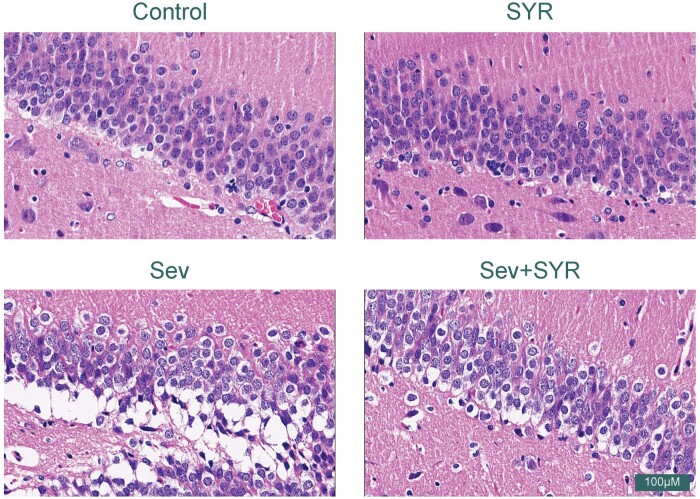
Representative images of hippocampal tissue damage showing marked vacuolation and nuclear pyknosis of neurons in the Sev group. H&E staining.

H&E staining of hippocampal tissue sections revealed distinct morphological differences across the treatment groups (Fig. 2). In the control group, neuronal cells within the hippocampus showed regular, dense nuclear staining, and the cytoplasm was evenly distributed, reflecting normal histological architecture. SYR treatment alone did not cause any noticeable histopathological changes in the hippocampal structure compared to the control. In contrast, the Sev group exhibited pronounced neuronal damage, characterized by nuclear condensation, vacuolization, disarrayed arrangement, and general tissue loosening, indicating cytoplasmic shrinkage. These changes are indicative of neurodegenerative alterations commonly associated with sevoflurane-induced cognitive dysfunction. Remarkably, cotreatment with SYR and Sev appeared to attenuate these pathological alterations. The hippocampal neurons in the Sev+SYR group showed less nuclear condensation and reduced vacuolization and the overall hippocampal architecture seemed more preserved compared to the Sev-only group. This suggests that SYR has a protective effect against Sev-induced hippocampal damage, consistent with the hypothesis that SYR could mitigate neurodegeneration and support neuronal integrity in the context of sevoflurane exposure.

### SYR attenuates sevoflurane-induced neuroinflammation

**Figure 3.**
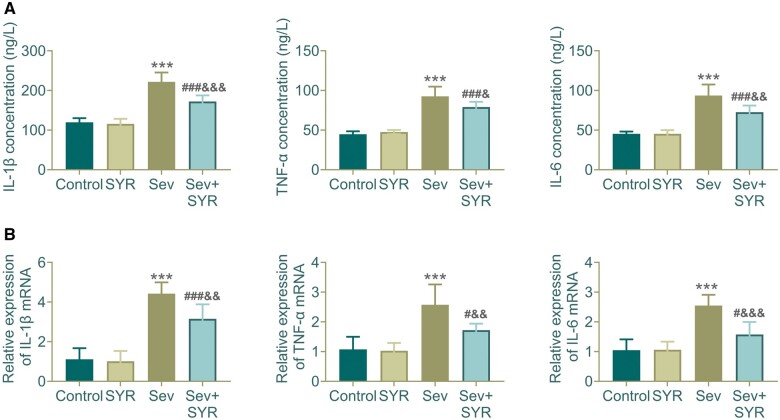
Cytokine quantification in hippocampal tissue of treated rodent groups. **(A)** Concentrations of IL-1β, TNF-α, and IL-6 were measured by ELISA. **(B)** Relative mRNA expression levels for the same cytokines determined by qPCR further confirm the anti-inflammatory effects of Sev+SYR treatment; n = 6, ^***^p < .001 compared to the control group, ^#^p < .05, ^###^p < .001 compared to the Sev group; ^&^p < .05, ^&&^p < .01, ^&&&^p < .001 compared to the SYR group.

The expression of IL-1β, TNF-α, and IL-6 were detected by ELISA. In the Sev group, there was a significant upregulation of IL-1β, TNF-α, and IL-6 levels compared to the control, suggesting that sevoflurane exposure induces a strong inflammatory response. The SYR treatment alone did not significantly alter the cytokine levels from the control group. However, when SYR was administered in conjunction with Sev (Sev+SYR group), there was a notable decrease in the levels of these cytokines, indicating the anti-inflammatory potential of SYR in the context of Sev-induced neuroinflammation (Fig. 3A). Figure 3B shows the relative gene expression levels of the same cytokines as determined by qPCR. Consistent with the ELISA data, the mRNA expression levels of IL-1β, TNF-α, and IL-6 were markedly elevated in the Sev group, reinforcing the notion of an inflammatory reaction. Similarly, SYR treatment in the Sev+SYR group significantly reduced the expression of these genes, further corroborating the anti-inflammatory effects of SYR observed in the ELISA data. The combined findings from ELISA and PCR analyses suggest that sevoflurane induces a pronounced inflammatory response in the hippocampus, which can be mitigated by SYR treatment. These anti-inflammatory properties of SYR may contribute to its neuroprotective effects against sevoflurane-induced cognitive dysfunction.

### SYR attenuates sevoflurane-induced glial cell activation

**Figure 4.**
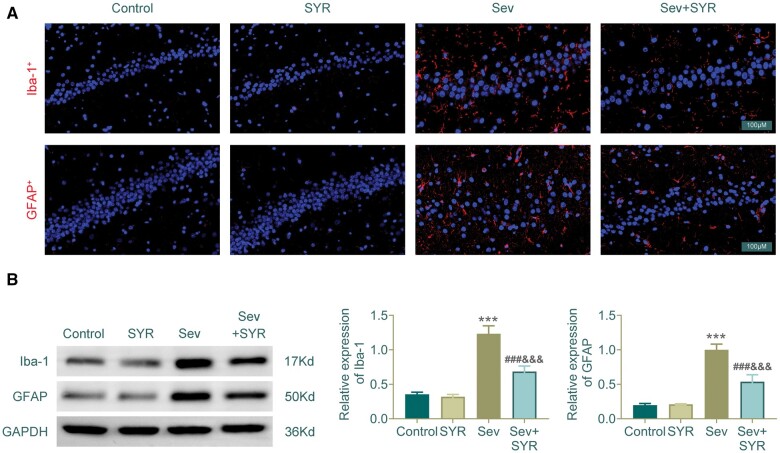
Immunofluorescence and Western-blot analysis of glial markers in hippocampal sections. **(A)** Immunofluorescent staining for Iba-1 and GFAP. **(B)** Western-blot bands for Iba-1 and GFAP, with densitometric analysis showing relative expression levels. GAPDH is used as a loading control. Scale bar in immunofluorescence images represents 100 µm; n = 6, ^***^p < .001 compared to the control group, ^###^p < .001 compared to the Sev group, ^&&&^p < .001 compared to the SYR group.

In the assessment of microglial activation, immunofluorescence analysis demonstrated a significant upregulation of Iba-1 in the Sev group when compared to the control, as indicated by the enhanced red fluorescence. This effect was mitigated in the Sev + SYR group, suggesting that SYR treatment attenuated the sevoflurane-induced microglial activation (Fig. 4A). Similarly, astrocyte activation was assessed by GFAP expression. The Sev group exhibited a marked increase in GFAP, shown by the increased red fluorescence, which was notably reduced in the Sev + SYR group, indicating a protective effect of SYR against astrocyte activation induced by sevoflurane (Fig. 4A). Western-blot analysis corroborated these findings, with the Sev group displaying elevated levels of Iba-1 and GFAP proteins compared to the control, implying activation of microglia and astrocytes. Treatment with SYR, particularly in the Sev + SYR group, was associated with a reduction in the expression of both Iba-1 and GFAP, supporting the immunofluorescence results. Quantification of the bands confirmed the visual observations, with a significant decrease in the relative expression of Iba-1 and GFAP in the Sev + SYR group in contrast to the Sev group (Fig. 4B). This suggests that SYR effectively mitigates glial cell activation following sevoflurane exposure.

### SYR attenuates Sev-induced apoptosis

**Figure 5.**
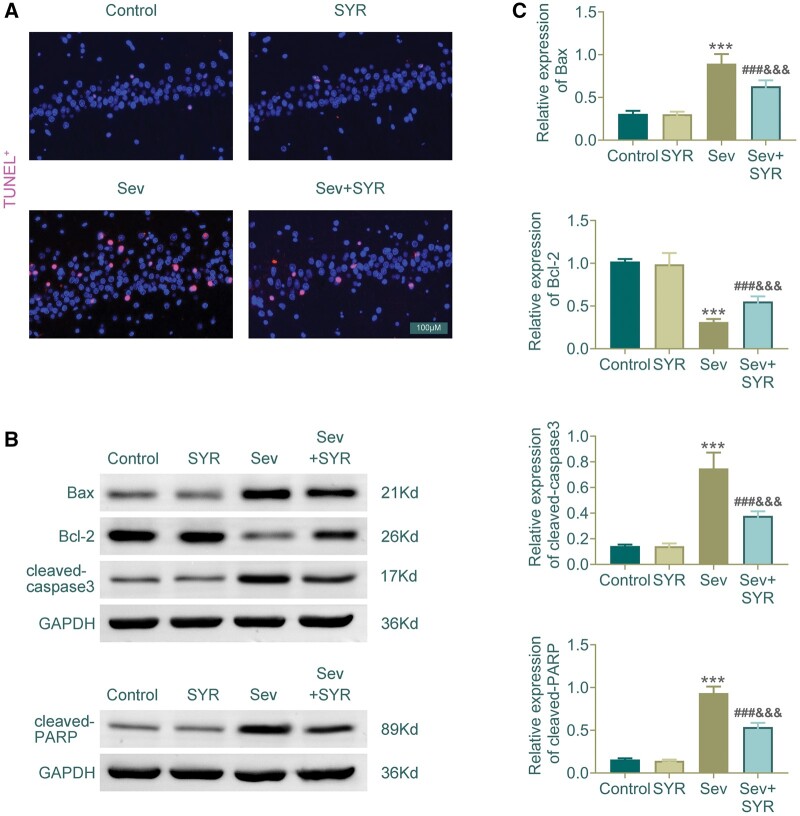
Detection of apoptosis in hippocampal tissue and analysis of apoptotic protein markers. **(A)** TUNEL staining indicates apoptotic cells, with the Sev group showing increased apoptosis, which is mitigated in the Sev+SYR group. **(B)** Western-blot analysis for apoptotic markers demonstrates the expression levels of Bax, Bcl-2, cleaved-caspase3, and cleaved-PARP. **(C)** The densitometric quantification shows a significant decrease in proapoptotic Bax and cleaved-caspase3 levels and an increase in antiapoptotic Bcl-2 in the Sev+SYR group compared to Sev, suggesting a protective effect of SYR cotreatment against Sev-induced apoptosis. GAPDH serves as a loading control. Scale bar in TUNEL images represents 100 µm; n = 6, ^***^p < .001 compared to the control group, and ^###^p < .001 compared to the Sev group, ^&&&^p < .001 compared to the SYR group.

The evaluation of apoptotic activity through TUNEL staining revealed a substantial increase in cell apoptosis within the hippocampal tissue of the Sev group, as evidenced by the increased number of TUNEL-positive cells, depicted by the bright pink fluorescence. The Sev + SYR group, however, showed a marked reduction in TUNEL-positive cells, suggesting that SYR treatment attenuates the apoptotic effects induced by sevoflurane (Fig. 5A). Western-blot analysis provided further insight into the apoptotic pathways. A significant upregulation of proapoptotic Bax and cleaved-caspase3 was observed in the Sev group compared to the control, alongside a downregulation of antiapoptotic Bcl-2, indicative of enhanced apoptotic signaling. The addition of SYR in the Sev + SYR group led to a decrease in Bax and cleaved-caspase3 levels and an increase in Bcl-2, as shown by the band intensity in comparison to the Sev group (Fig. 5B). Cleaved-PARP, a substrate of caspase-3 and an indicator of apoptosis, followed a similar pattern with its expression being significantly higher in the Sev group and reduced in the Sev+SYR group (Fig. 5B). The quantification of protein expression levels confirmed these observations, where the Sev group demonstrated an increase in the proapoptotic markers and a decrease in the antiapoptotic marker, which were significantly reversed upon SYR treatment in the Sev + SYR group (Fig. 5C). These results suggest that SYR exerts a neuroprotective effect by modulating apoptotic signaling pathways in hippocampal tissues exposed to sevoflurane.

### SYR regulates the SIRT1/Tau pathway

**Figure 6.**
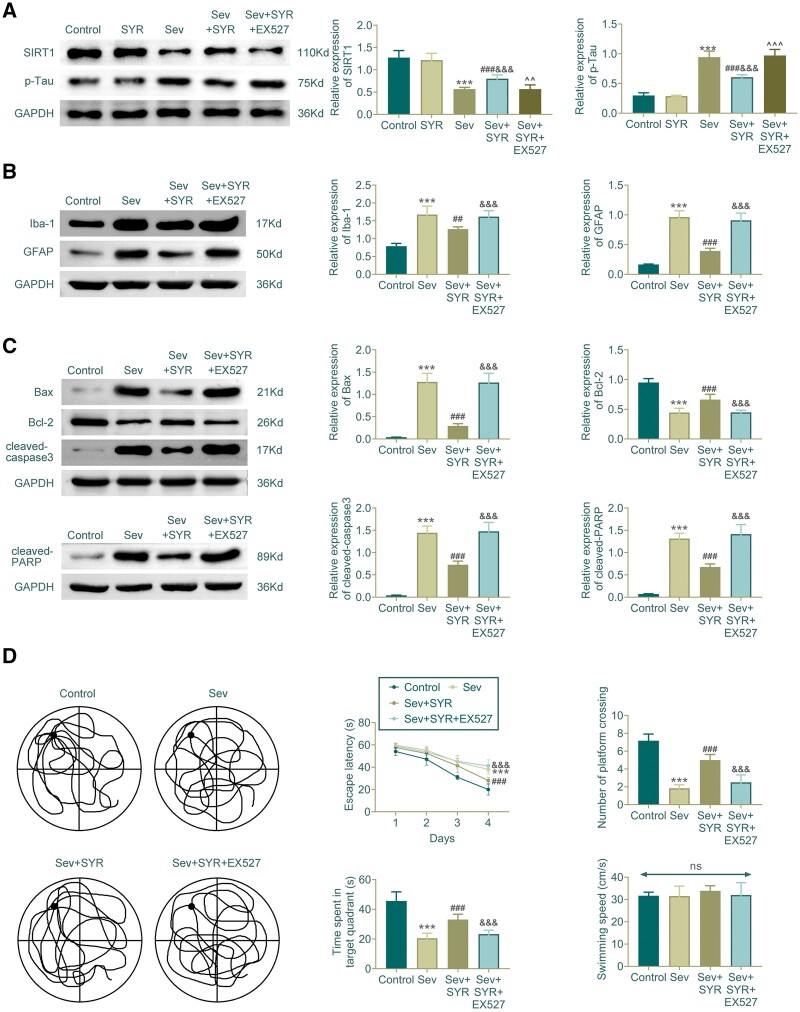
Multifaceted evaluation of the neuroprotective effects of SYR and the impact of SIRT1 inhibition in aged SD rats. **(A)** Western-blot analysis shows levels of SIRT1 and p-Tau. SYR treatment reduces Tau phosphorylation via SIRT1 modulation, which is reversed by the SIRT1 inhibitor EX527. **(B)** The expression of glial markers Iba-1 and GFAP, as determined by Western blot, is downregulated in the Sev+SYR group, indicating reduced gliosis. This effect is abrogated by EX527. **(C)** Analysis of apoptotic markers Bax, Bcl-2, cleaved-caspase3, and cleaved-PARP reveals SYR’s protective effect against Sev-induced apoptosis, which is mitigated by EX527. **(D)** Behavioral assessments through the water maze test demonstrate improved escape latency, number of platform crossings, and time spent in the target quadrant with SYR treatment, signifying enhanced cognitive function. SIRT1 inhibition by EX527 negates these improvements, suggesting that SYR’s beneficial effects are SIRT1-dependent. Swimming speed remains unaffected across all groups, indicating motor function is not compromised. Each group consists of 6 aged SD rats; n = 6, ^***^p < .001 compared to the control group, ^###^p < .001 compared to the Sev group, and ^&&&^p < .001 compared to the Sev+SYR group.

Western-blot analysis demonstrated the expression levels of SIRT1 and p-Tau in the hippocampal tissue of the different experimental groups. In the Sev group, a reduction in SIRT1 expression and an increase in p-Tau phosphorylation were observed compared to the control. Treatment with Sev along with SYR showed a marked increase in SIRT1 levels and a decrease in p-Tau, suggesting the involvement of SYR in modulating Tau phosphorylation through SIRT1. The addition of EX527, a SIRT1 inhibitor, reversed the effects of SYR on SIRT1 and p-Tau, underscoring the role of SIRT1 in the protective effects of SYR against Tau hyperphosphorylation induced by sevoflurane (Fig. 6A). It also showed an increase in glial activation markers Iba-1 and GFAP in the Sev group, which was reduced by SYR treatment. This reduction was not observed with the addition of EX527, suggesting that the anti-inflammatory effects of SYR are mediated through SIRT1 (Fig. 6B). Similarly, proapoptotic Bax and cleaved-caspase3 levels were elevated in the Sev group and reduced with SYR treatment. This effect was reversed by EX527, while antiapoptotic Bcl-2 showed an opposite trend. Cleaved-PARP levels followed a similar pattern, further confirming the role of SIRT1 in SYR’s action (Fig. 6C). Behavioral assessment using the MWM indicated that Sev exposure impaired cognitive function, evidenced by increased escape latency and reduced number of platform crossings. Treatment with Sev along with SYR improved these parameters, which was negated by the concurrent administration of EX527, indicating that SYR enhances cognitive function via the SIRT1 pathway (Fig. 6D). In summary, SYR attenuated cognitive deficits, glial activation, and apoptotic signaling induced by Sev, and these effects were mediated by the SIRT1 pathway, as the inhibition of SIRT1 by EX527 abrogated the protective effects of SYR.

## DISCUSSION

The present study offers novel insights into the therapeutic potential of SYR in mitigating cognitive dysfunction induced by Sev in rats, thereby expanding our understanding of neuroprotective strategies against anesthesia-induced cognitive deficits. Our findings reveal that SYR significantly alleviates cognitive impairments caused by Sev, as evidenced by the modified MWM test results. The Sev group displayed extended escape latency, while the Sev+SYR group showed substantial improvement, closely mirroring the performance of the control group. This improvement was not attributable to motor ability changes, suggesting a direct effect of SYR on cognitive functions. Moreover, our histological analysis of hippocampal tissues revealed that SYR exerts a protective influence against Sev-induced neurodegeneration. Similarly, a study found that the combination of chlorogenic acid and (+)-syringaresinol-di-O-β-D-glucoside significantly increased hippocampal BDNF protein expression in rats, suggesting a neuroprotective role through enhancement of cognitive and emotional regulation via hippocampal pathways (9). This further supports the potential of compounds like SYR in safeguarding neuronal integrity and cognitive function (9, 10). The Sev group exhibited notable neuronal damage, such as nuclear condensation and vacuolization, hallmarks of neurodegenerative alterations. In contrast, SYR cotreatment appeared to preserve hippocampal architecture and mitigate these pathological changes, underscoring its potential in safeguarding neuronal integrity. This is in line with the study by Naomi et al., which highlights that dietary polyphenols, like those in SYR, can protect against cognitive decline and neurodegeneration. These polyphenols act through various mechanisms, such as combating neuroinflammation, oxidative stress, and mitochondrial damage, thereby offering a potential therapeutic approach for cognitive impairments and related neurodegenerative conditions (11).

A critical aspect of our study is the investigation into the role of SYR in modulating neuroinflammatory responses to Sev. Our results, supported by both ELISA and qPCR analyses, demonstrate a significant reduction in proinflammatory cytokines (IL-1β, TNF-α, and IL-6) following SYR treatment in Sev-exposed rats. This anti-inflammatory action of SYR is a pivotal finding, suggesting its utility in dampening the inflammatory cascades associated with cognitive dysfunction. This is corroborated by studies that have found elevated levels of these cytokines in conditions like depression, Alzheimer disease, and OCD, where they are associated with cognitive impairments. Therefore, the reduction of these cytokines by SYR could be instrumental in mitigating cognitive dysfunction related to neuroinflammation (12–14).

Additionally, our study delves into the effects of SYR on glial cell activation, a key element in neuroinflammatory processes. Immunofluorescence and Western-blot analyses indicate that SYR treatment effectively attenuates the Sev-induced activation of microglia and astrocytes. This modulation of glial response could be instrumental in mitigating the neuroinflammatory milieu conducive to cognitive decline. In line with this, the study by Tian et al. reveals that sevoflurane activates microglial caspase-1, leading to pyroptosis and progression of AD-related pathology, highlighting the pivotal role of microglial and astrocytic responses in neurodegenerative processes. Therefore, the ability of SYR to modulate these glial cell activities presents a promising therapeutic approach for addressing the underlying neuroinflammatory mechanisms contributing to cognitive impairment (15).

Our investigation into apoptotic pathways reveals that SYR significantly countered Sev-induced apoptosis in hippocampal tissues. The reduction in apoptotic markers and the reversal of pro- and antiapoptotic protein expressions in the Sev+SYR group highlight SYR’s potential in curbing neuronal cell death. The histopathological and molecular data indicate that SYR mitigates sevoflurane-induced neuronal damage, inflammation, glial activation, and apoptosis. These results suggest a protective effect of SYR rather than a regenerative one, as we did not observe a significant increase in markers that are typically associated with neurogenesis or cell regeneration. It is more likely that SYR prevents further degeneration and supports the survival of existing neurons, rather than promoting the regeneration of new cells after neuronal loss. This is corroborated by the study, which found that sevoflurane exposure causes neuronal apoptosis and cognitive dysfunction by inducing endoplasmic reticulum (ER) stress via IP3R activation in aged rats and isolated hippocampal neurons (16). The ability of SYR to modulate these pathways and prevent neuronal apoptosis underscores its potential as a neuroprotective agent against sevoflurane-induced neurotoxicity (17).

A remarkable aspect of our findings is the elucidation of the role of SYR in the SIRT1/Tau pathway (18, 19). The modulation of Tau phosphorylation through SIRT1 signifies a deeper mechanistic understanding of neuroprotective effects. SYR protection against sevoflurane-induced cognitive dysfunction, neuroinflammation, and apoptosis may be mediated through the modulation of the SIRT1 activity. The precise molecular interactions of SIRT1 with these various pathways warrant further investigation but we propose that the anti-inflammatory and antiapoptotic effects of SYR are likely due to its influence on SIRT1 regulation of transcription factors and apoptotic proteins. This is supported by studies that demonstrate the critical role of Tau protein in cognitive dysfunction caused by anesthetic drugs and the regulatory role of SIRT1 in Tau phosphorylation. These studies highlight that activation of SIRT1 and modulation of Tau phosphorylation can effectively alleviate cognitive dysfunction induced by anesthesia, further underscoring the potential of SYR in targeting these pathways for neuroprotection (4, 20, 21). The intervention with EX527, a SIRT1 inhibitor, further confirms the pivotal role of SIRT1 in mediating SYR’s protective actions against Sev-induced cognitive deficits, glial activation, and apoptotic signaling.

There are limitations to this study. These include the importance of examining other cerebral regions that might also be susceptible to sevoflurane exposure, such as the cortex, amygdala, and cerebellum, to obtain a comprehensive understanding of the anesthetic’s neuropathological impact. In addition, the current study focused on the hippocampus due to its established sensitivity to anesthetics and relevance to the behavioral assay. In our study of hippocampal histology, analyses of the subiculum and entorhinal cortex were not included. Furthermore, while our study did not directly investigate the metabolic pathways of sevoflurane or its clearance mechanisms in relation to SYR administration, the neuroprotective effects observed suggest that SYR might influence the cellular response to sevoflurane exposure. There are a few reports suggesting that derivatives of SYR are potent modulators of adipogenesis and glucose consumption; these may be the subject of further studies for the prevention and treatment of disorders of lipid and glucose metabolism (22). SYR is known for its antioxidant properties and its ability to enhance the efficiency of mitochondrial function, which could indirectly aid in the detoxification processes. Enhanced mitochondrial function may improve cellular resilience to stress, potentially influencing the metabolism of anesthetic agents. Additionally, SYR modulation of SIRT1 may also play a role in cellular homeostasis and the management of xenobiotic stress, which could implicitly affect sevoflurane’s cellular handling.

In conclusion, our study sheds light on the multifaceted neuroprotective mechanisms of SYR against Sev-induced cognitive dysfunction, encompassing anti-inflammatory, antiapoptotic, and Tau phosphorylation modulation pathways (23). These findings not only contribute to a better understanding of anesthesia-related cognitive impairments but also open avenues for developing targeted therapeutic interventions. Future research should aim to elucidate the molecular underpinnings of SYR’s effects and explore its clinical applicability in preventing or treating POCDs. The present results suggest potential clinical applications of SYR in improving postoperative cognitive outcomes, highlighting its significance in perioperative medicine.

## Data Availability

All data generated or analyzed during this study are included in this published article. The datasets used and/or analyzed during this study are available from the corresponding author on reasonable request.
